# Sociodemographic Characteristics and Mental and Physical Health Diagnoses of Yazidi Refugees Who Survived the Daesh Genocide and Resettled in Canada

**DOI:** 10.1001/jamanetworkopen.2023.23064

**Published:** 2023-07-12

**Authors:** Nour Hassan, Annalee Coakley, Ibrahim Al Masri, Rachel Talavlikar, Michael Aucoin, Rabina Grewal, Adl K. Khalaf, Shahla Murad, Kerry A. McBrien, Paul Ronksley, Gabriel E. Fabreau

**Affiliations:** 1 Department of Medicine, Cumming School of Medicine, University of Calgary, Calgary, Alberta, Canada; 2 O’Brien Institute for Public Health, Cumming School of Medicine, University of Calgary, Calgary, Alberta, Canada; 3 Department of Family Medicine, Cumming School of Medicine, University of Calgary, Calgary, Alberta, Canada; 4 Mosaic Refugee Health Clinic, Mosaic Primary Care Network, Calgary, Alberta, Canada; 5 Department of Community Health Sciences, Cumming School of Medicine, University of Calgary, Calgary, Alberta, Canada; 6 Department of English Language, College of Education for Human Sciences, University of Mosul, Mosul, Iraq; 7 Independent scholar

## Abstract

**Importance:**

The health status of Yazidi refugees, a group of ethnoreligious minority individuals from northern Iraq who resettled in Canada between 2017 and 2018 after experiencing genocide, displacement, and enslavement by the Islamic State (Daesh), is unknown but important to guide health care and future resettlement planning for Yazidi refugees and other genocide victims. In addition, resettled Yazidi refugees requested documentation of the health impacts of the Daesh genocide.

**Objective:**

To characterize sociodemographic characteristics, mental and physical health conditions, and family separations among Yazidi refugees who resettled in Canada.

**Design, Setting, and Participants:**

This retrospective clinician- and community-engaged cross-sectional study included 242 Yazidi refugees seen at a Canadian refugee clinic between February 24, 2017, and August 24, 2018. Sociodemographic and clinical diagnoses were extracted through review of electronic medical records. Two reviewers independently categorized patients’ diagnoses by *International Statistical Classification of Diseases, Tenth Revision, Clinical Modification* (*ICD-10-CM*) codes and *ICD-10-CM* chapter groups. Diagnosis frequencies were calculated and stratified by age group and sex. Five expert refugee clinicians used a modified Delphi approach to identify diagnoses likely to be associated with Daesh exposure, then corroborated these findings with Yazidi leader coinvestigators. A total of 12 patients without identified diagnoses during the study period were excluded from the analysis of health conditions. Data were analyzed from September 1, 2019, to November 30, 2022.

**Main Outcomes and Measures:**

Sociodemographic characteristics; exposure to Daesh captivity, torture, or violence (hereinafter, Daesh exposure); mental and physical health diagnoses; and family separations.

**Results:**

Among 242 Yazidi refugees, the median (IQR) age was 19.5 (10.0-30.0) years, and 141 (58.3%) were female. A total of 124 refugees (51.2%) had direct Daesh exposure, and 60 of 63 families (95.2%) experienced family separations after resettlement. Among 230 refugees included in the health conditions analysis, the most common clinical diagnoses were abdominal and pelvic pain (47 patients [20.4%]), iron deficiency (43 patients [18.7%]), anemia (36 patients [15.7%]), and posttraumatic stress disorder (33 patients [14.3%]). Frequently identified *ICD-10-CM* chapters were symptoms and signs (113 patients [49.1%]), nutritional diseases (86 patients [37.4%]), mental and behavioral disorders (77 patients [33.5%]), and infectious and parasitic diseases (72 patients [31.3%]). Clinicians identified mental health conditions (74 patients [32.2%]), suspected somatoform disorders (111 patients [48.3%]), and sexual and physical violence (26 patients [11.3%]) as likely to be associated with Daesh exposure.

**Conclusions and Relevance:**

In this cross-sectional study, Yazidi refugees who resettled in Canada after surviving the Daesh genocide experienced substantial trauma, complex mental and physical health conditions, and nearly universal family separations. These findings highlight the need for comprehensive health care, community engagement, and family reunification and may inform care for other refugees and genocide victims.

## Key Points

**Question:**

What are the sociodemographic characteristics, mental and physical health conditions, and family separation experiences of Yazidi refugees who resettled in Canada?

**Findings:**

In this cross-sectional study of 242 Yazidi refugees who survived genocide and received care at a specialized Canadian refugee clinic, 51.2% were directly exposed to Daesh captivity, torture, or violence, and 95.2% experienced family separations. Yazidi refugees presented with physical symptoms and signs (49.1%), nutritional diseases (37.4%), mental and behavioral disorders (33.5%), and infectious and parasitic diseases (31.3%), and expert refugee clinicians suspected nearly one-half (48.3%) of refugees had somatoform disorders.

**Meaning:**

These findings suggest that resettled Yazidi refugees are highly traumatized, have clinically complex health conditions, and require holistic care that attends to both mental and physical health.

## Teaser

This cross-sectional study characterizes sociodemographic characteristics, mental and physical health conditions, and family separations among Yazidi refugees who resettled in Canada.

## Introduction

The Yazidi ethnoreligious minority group, with an estimated global population of 800 000 individuals who are predominantly from northern Iraq, has experienced decades of persecution.^1,2^ In August 2014, the self-proclaimed Islamic State, known as Daesh, targeted approximately 400 000 Yazidi individuals in the Sinjar province of Iraq for mass displacement, execution, systemic rape, and enslavement, leading to approximately 5500 deaths and 7000 kidnappings, with captives held for months to years.^3,4,5,6^ The United Nations labeled these atrocities a genocide.^3^ Daesh systematically executed male individuals older than 12 years, separated families according to age and sex, used boys as child soldiers, and left hundreds of families decimated or displaced.^6^ Daesh captured women and girls and subjected them to forced marriage, slavery, and sexual and gender-based violence, including rape and beating.^6,7^ Many Yazidi individuals, including children, witnessed family members being tortured and/or killed.^6,8^ Many nuclear families perished entirely. Today, many family members of Yazidi refugees remain in internally displaced peoples (IDP) camps, in captivity, or missing.^3,8^

Between 2017 and 2018, Canada resettled approximately 1500 Yazidi refugees, predominantly women and children, through its Survivors of Daesh program.^5,8^ Resettlement occurred shortly after rescue, unlike the experiences of many refugees who spend approximately 7 to 9 years in refugee camps before resettlement.^8^ Yazidi refugees presented health care professionals with unanticipated challenges due to the acute trauma and complex health issues that required more intensive and specialized health care services.^9,10^ These challenges were compounded by traumatic family separations, known to worsen the health and well-being of refugees after resettlement.^8,11,12,13^

Several studies have identified the long-term and detrimental health impacts of surviving genocide, including posttraumatic stress disorder (PTSD), depression, and anxiety, in conflict areas such as Rwanda and Bosnia.^14,15,16,17,18^ Current research has primarily investigated the mental health needs of displaced Yazidi refugees in Iraq or Turkey, revealing complex psychosocial needs and high rates of PTSD and major depression.^19,20,21,22,23,24,25^ While physical injuries and communicable and noncommunicable diseases have been reported among Yazidi refugees in camps,^22^ data on the physical health of Yazidi refugees who resettled in high-income countries are scarce. Furthermore, somatic symptoms associated with trauma have been documented among traumatized refugees and survivors of sexual violence^26,27,28,29,30^; however, only 1 German study reported somatic symptoms associated with exposure to Daesh captivity, torture, or violence (hereinafter, Daesh exposure) among female Yazidi refugees.^31,32^ Thus, somatoform disorders among Yazidi refugees remain poorly studied.

Given that the complex health needs of Yazidi refugees have presented challenges to health care professionals,^10,33,34,35^ a comprehensive postresettlement health assessment is needed. Importantly, this study originated from the requests of Yazidi patients and their recognized need to document the health impacts of the Daesh genocide. Thus, we performed a clinician- and community-engaged retrospective cross-sectional study to investigate sociodemographic characteristics, mental and physical health conditions, and family separations among Yazidi refugees in Canada. In consideration of the inherent limitations of clinical diagnoses derived from electronic medical records (EMRs),^36,37,38,39^ we consulted expert refugee clinicians (A.C., I.A.M., R.T., M.A., and G.E.F.) to identify conditions likely to be associated with Daesh exposure. These findings were further interpreted and validated by 2 Yazidi leader coinvestigators (A.K.K. and S.M).

## Methods

### Study Design, Setting, and Participants

We conducted a retrospective clinician- and community-engaged cross-sectional study of 242 Yazidi refugees who resettled in an urban Canadian center. All participants visited a specialized refugee health care clinic within 1 year of arrival between February 24, 2017, and August 24, 2018. Data were analyzed from September 1, 2019, to November 30, 2022. This study was approved by the University of Calgary Conjoint Health Research Ethics Board with a waiver of informed consent due to the retrospective nature of the study and minimal risks to participants. The study followed the Strengthening the Reporting of Observational Studies in Epidemiology (STROBE) reporting guideline for cross-sectional studies.

The specialized health care clinic provides refugees and asylum claimants with primary and multispecialty care services, including children and family care, women’s health care, mental health care, health education, and social services. Nearly all locally resettled Yazidi refugees (242 of 245 [98.8%]) were estimated to have visited the clinic.^8^ We extracted data from EMRs via review using the keyword *Yazidi* or sociodemographic data that identified Kurmanji-speaking individuals from Iraq or Syria.

### Variables

#### Sociodemographic Characteristics

We extracted and manually verified sociodemographic data, including age at intake, biological sex, country of birth, marital status, spousal status, primary language spoken, number of children, educational level, English language proficiency, and refugee camp exposure.

#### Daesh Exposure

We used EMR keywords (eg, *kill*, *capture*, *torture*, *Daesh*, and *ISIS*) to assess exposure to Daesh captivity, torture, or violence. We defined direct Daesh exposure as being personally held captive, being enslaved, or experiencing traumatic events, including witnessing or experiencing firsthand violence. Indirect Daesh exposure was defined as not being personally held captive or witnessing violence but having family members who were captured, killed, missing, or tortured. We defined individuals without documentation of Daesh exposure as having unknown exposure and individuals with explicit documentation of no Daesh exposure as having no exposure.

#### Family Separation

We used a standardized data extraction protocol to collect family separation data, grouped into family units, as follows: (1) keyword searches were used for separated family members, (2) advocacy letters requesting support to reunify individuals’ family members were identified, and (3) individuals were assumed to be separated from family if they or a family member were captured by Daesh. We classified family separations as either nuclear, nonnuclear, or both nuclear and nonnuclear. We used the government of Canada’s definition of a nuclear family, which includes an individual’s spouse or common-law partner, parents, and dependent children younger than 18 years.^40^

#### Clinical Data

Two reviewers (I.A.M. and R.G.) independently extracted clinical data, including medical diagnoses, treatment information, and medical histories. The reviewers resolved diagnostic disagreements by consensus or with involvement of a third senior investigator (G.E.F.). Next, reviewers assigned an *International Statistical Classification of Diseases, Tenth Revision, Clinical Modification* (*ICD-10-CM*) code to each diagnosis, then grouped individual diagnostic codes into *ICD-10-CM* chapter subgroups.^41,42^

### Clinician Consensus-Building Procedure

To supplement our *ICD-10-CM* analysis, we incorporated a clinician consensus-building procedure to identify specific health conditions that were likely to be associated with Daesh trauma. This approach was necessitated by the diagnostic ambiguity surrounding trauma-related conditions like somatoform disorders, which are often undetected by stand-alone EMR-based analyses,^36,37,38,39^ and the limited clinic duration for Yazidi refugees at the time of study initiation, which was insufficient to fully establish these conditions. We used a modified online Delphi method, which is commonly used to develop clinical practice guidelines, for 3 rounds of voting, discussion, and consensus building.^43,44^ We purposively selected 5 clinician experts (A.C., I.A.M., R.T., M.A., and G.E.F.) with 52 years of combined experience in refugee clinical care, including Yazidi patients.

In round 1, clinicians independently reviewed a list of all conditions found in the Yazidi cohort and selected conditions they perceived to be associated with Daesh exposure and those they suspected were somatoform disorders not otherwise explained. Clinicians labeled these conditions as suspected somatoform disorders to acknowledge some inherent diagnostic uncertainty and explained their selections using open-text boxes. In round 2, the moderator (N.H.) presented a vote summary to clinicians and set an a priori consensus cutoff of 80% (4 of 5 votes) for inclusion in the list of conditions suspected to be associated with Daesh exposure. Conditions receiving 0 or 1 vote were excluded, while those with 2 or 3 votes were discussed until clinicians reached consensus. In round 3, the moderator provided a revised list of conditions suspected to be associated with Daesh exposure. Clinicians discussed them until unanimous agreement and grouped them into 3 groups of clinically relevant conditions suspected to be associated with Daesh exposure. Following community-engaged research principles,^45,46^ we presented plain language summaries to 2 Yazidi community leaders (A.K.K. and S.M., one of whom resettled in Canada and one of whom resettled in an IDP camp in Iraq) to verify the relevance of these clinical groupings to their communities and finalize their description and interpretation. The 2 Yazidi leaders contextualized variables (eg, family separation), validated definitions (eg, Daesh exposure), and interpreted results.^45,46,47^

### Statistical Analysis

We stratified the clinical groups by age (0-11 years, 12-17 years, and ≥18 years) and sex (among adults only) and used a χ^2^ test to compare their prevalence among Yazidi subgroups. Continuous variables were reported as medians and IQRs given their skewed distribution, and *ICD-10-CM* diagnoses and chapters were summarized as frequencies and proportions. The significance threshold was 2-tailed *P* < .05. We used Stata/IC software, version 16 (StataCorp LLC), for all analyses.

## Results

### Sociodemographic Characteristics

Among 242 Yazidi refugees, the median (IQR) age was 19.5 (10.0-30.0) years (range, 6 months to 74 years). Of those, 141 refugees (58.3%) were female, 101 (41.7%) were male, 130 (53.7%) were adults, 72 (29.8%) were children aged 6 months to 11 years, and 40 (16.5%) were adolescents aged 12 to 17 years (Table 1). Yazidi refugees primarily spoke Kurdish Kurmanji (234 [96.7%]) but did not speak English (239 [98.8%]), and many adults lacked formal education (58 of 130 [44.6%]). A total of 124 refugees (51.2%) had direct Daesh exposure, among whom 52 (41.9%) were female. We identified 63 families, with a mean of 3.8 members per family (range, 1-8 members). Among families, 60 (95.2%) reported family separations; of those, 37 (61.7%) reported separation from both nuclear and nonnuclear family members, and 23 (38.3%) reported separation from nuclear family members only. No families reported separation from nonnuclear family members alone. Among 130 married adults, 25 (19.2%) reported their spouse was either captive, missing, or dead.

**Table 1.** Sociodemographic Characteristics of Yazidi Refugees at a Specialized Refugee Clinic in Canada

Characteristic	Participants, No. (%)				
	Overall (N = 242)	Children aged 6 mo to 11 y (n = 72)	Adolescents aged 12 to 17 y (n = 40)	Female adults aged ≥18 y (n = 82)	Male adults aged ≥18 y (n = 48)
Age at intake, median (IQR), y	19.5 (10.0-30.0)	5.0 (3.0-8.0)	14.0 (13.0-16.0)	31 (25.0-42.0)	28.5 (22.5-32.5)
Sex					
Female	141 (58.3)	41 (56.9)	18 (45.0)	NA	NA
Male	101 (41.7)	31 (43.1)	22 (55.0)	NA	NA
Country of citizenship					
Iraq	235 (97.1)	72 (100)	39 (97.5)	80 (97.6)	44 (91.7)
Syria	7 (2.9)	0	1 (2.5)	2 (2.4)	4 (8.3)
English language proficiency					
None	239 (98.8)	72 (100)	38 (95.0)	81 (98.8)	48 (100)
Little or some	3 (1.2)	0	2 (5.0)	1 (1.2)	0
Primary language					
Arabic	2 (0.8)	0	0	1 (1.2)	1 (2.1)
Badini	5 (2.1)	1 (1.4)	2 (5.0)	1 (1.2)	1 (2.1)
Kurmanji	234 (96.7)	70 (97.2)	38 (95.0)	80 (97.6)	46 (95.8)
Unknown	1 (0.4)	1 (1.4)	0	0	0
Educational level					
No formal education	110 (45.5)	47 (65.3)	5 (12.5)	49 (59.8)	9 (18.8)
Less than high school	116 (47.9)	22 (30.6)	32 (80.0)	31 (37.8)	31 (64.6)
High school	8 (3.3)	NA	2 (5.0)	2 (2.4)	4 (8.3)
Postsecondary school	1 (0.4)	NA	NA	0	1 (2.1)
Unknown	7 (2.9)	3 (4.2)	1 (2.5)	0	3 (6.3)
Refugee camp exposure					
Yes	65 (26.9)	19 (26.4)	10 (25.0)	19 (23.2)	17 (35.4)
Unknown	177 (73.1)	53 (73.6)	30 (75.0)	63 (76.8)	31 (64.6)
Marital status					
Married	76 (31.4)	NA	NA	48 (58.5)	28 (58.3)
Single	156 (64.5)	NA	NA	24 (29.3)	20 (41.7)
Widowed	8 (3.3)	NA	NA	8 (9.8)	0
Divorced	2 (0.8)	NA	NA	2 (2.4)	0
Spousal status					
Missing	3 (1.2)	NA	NA	3 (3.7)	0
Held captive	14 (5.8)	NA	NA	13 (15.9)	1 (2.1)
Dead	8 (3.3)	NA	NA	8 (9.8)	0
Alive	61 (25.2)	NA	NA	34 (41.5)	27 (56.3)
NA	156 (64.5)	NA	NA	24 (29.3)	20 (41.7)
Number of children					
0	57 (23.6)	NA	NA	32 (39.0)	25 (52.1)
1-2	12 (5.0)	NA	NA	7 (8.5)	5 (10.4)
3-4	30 (12.4)	NA	NA	20 (24.4)	10 (20.8)
≥5	31 (12.8)	NA	NA	23 (28.0)	8 (16.7)
Exposure to Daesh captivity, torture, or violence					
Direct	124 (51.2)	38 (52.8)	25 (62.5)	52 (63.4)	9 (18.8)
Indirect	53 (21.9)	12 (16.7)	11 (27.5)	14 (17.1)	16 (33.3)
None	13 (5.4)	4 (5.6)	0	4 (4.9)	5 (10.4)
Unknown	52 (21.5)	18 (25.0)	4 (10.0)	12 (14.6)	18 (37.5)

Abbreviation: NA, not applicable.

### Clinical Diagnoses

**Figure 1.**
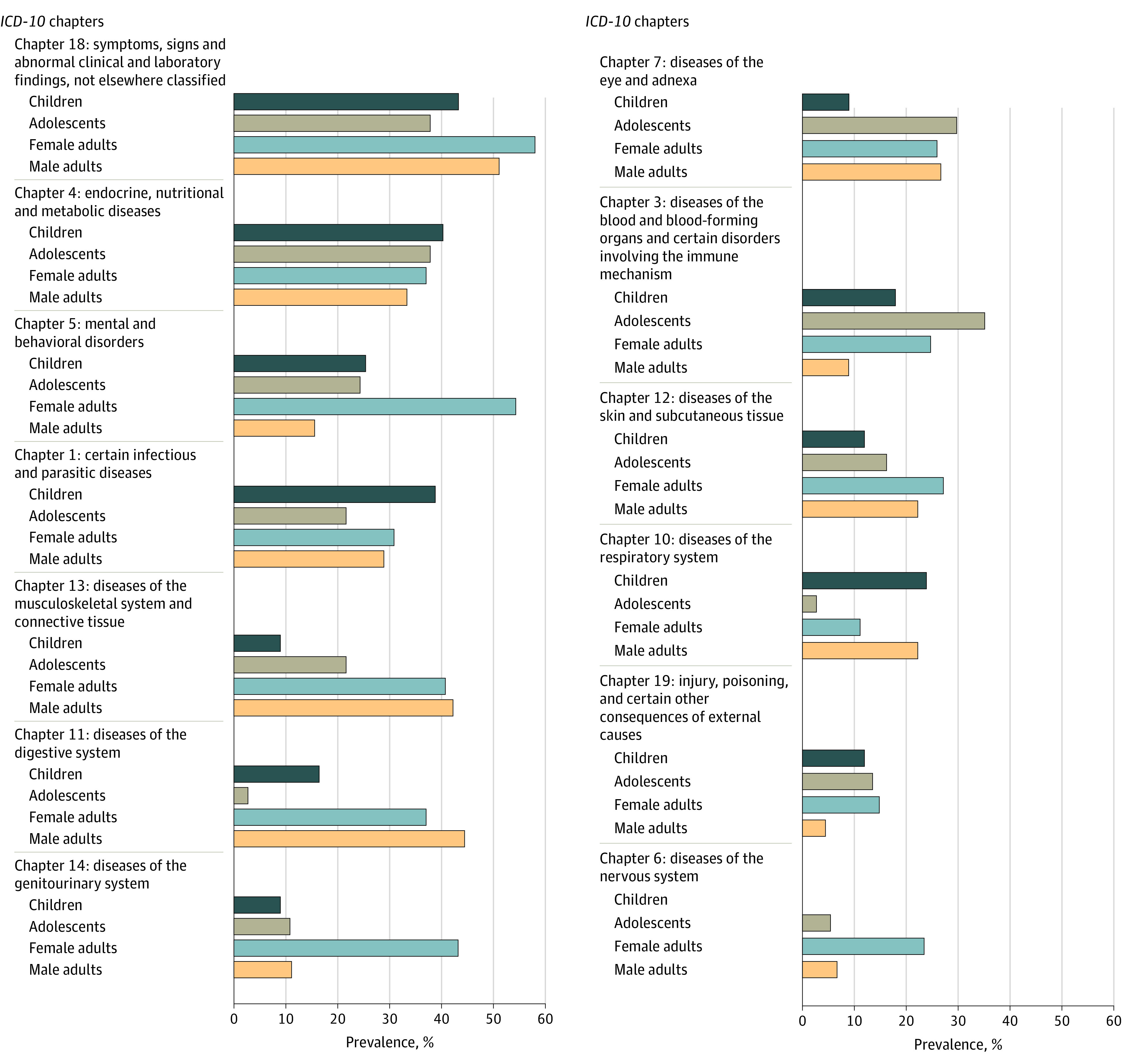
Prevalence of *International Statistical Classification of Diseases, Tenth Revision, Clinical Modification* (*ICD-10-CM*) Chapters Among Yazidi Refugees by Age and Sex Prevalence among 67 Yazidi children aged 6 months to 11 years, 37 adolescents aged 11 to 17 years, and 126 adults (81 women and 45 men) 18 years and older. Chapters are ranked from most prevalent (top) to least prevalent (bottom). Chapters prevalent among fewer than 10% of the entire cohort are not shown. Chapters are not mutually exclusive, and patients were counted once if they had more than 1 condition within each chapter.

A total of 12 patients without identified diagnoses during the study period were excluded from the health conditions analysis. Among the remaining 230 Yazidi patients, we identified a median (IQR) of 4 (2-6) diagnoses per patient (range, 1-41 diagnoses per patient). We recorded 1133 individual diagnoses comprising 282 unique *ICD-10-CM* diagnostic codes contained in 20 *ICD-10-CM *chapters. Patients’ clinic intake occurred within a median (IQR) of 8 (6-13) days after arrival in Canada, and the reported diagnoses were recorded within a median (IQR) of 12 (0-66) days after this initial clinic appointment. The most frequently identified *ICD-10-CM *chapters among 230 refugees were symptoms and signs (113 patients [49.1%]), nutritional diseases (86 patients [37.4%]), mental and behavioral disorders (77 patients [33.5%]), and infectious and parasitic diseases (72 patients [31.3%]). Additional data about the most common *ICD-10-CM *chapters and their frequencies by age group and sex are shown in Figure 1. The 5 most common diagnoses per *ICD-10-CM *chapter are shown in eTable 1 in Supplement 1.

Yazidi women had the highest observed prevalence of mental and behavioral disorders (44 of 81 patients [54.3%]), followed by children (17 of 67 patients [25.4%]), adolescents (9 of 37 patients [24.3%]), and men (7 of 45 patients [15.6%]). Adults had more musculoskeletal and connective tissue diseases (33 of 81 women [40.7%] and 19 of 45 men [42.2%]) than adolescents (8 of 37 patients [21.6%]) and children (6 of 67 patients [9.0%]). Children had a higher observed prevalence of infectious and parasitic diseases (26 of 67 patients [38.8%]) and respiratory diseases (16 of 67 patients [23.9%]) compared with other groups, while adolescents had more blood diseases (13 of 37 patients [35.1%]) and eye diseases (11 of 37 patients [29.7%]) (Figure 1). A heat map and illustration of the frequencies of *ICD-10-CM *chapters stratified by age group and sex are shown in the eFigure in Supplement 1.

**Figure 2.**
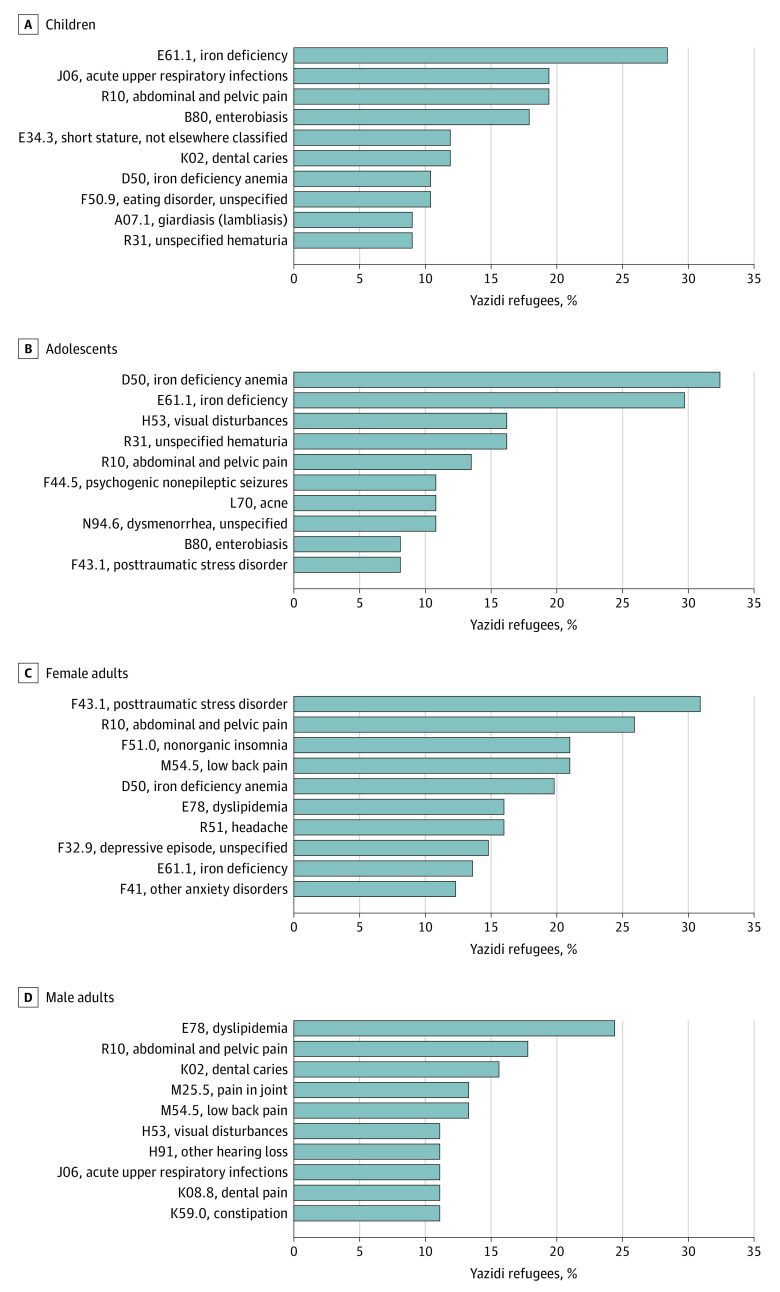
Ten Most Prevalent *International Statistical Classification of Diseases, Tenth Revision, Clinical Modification *Diagnoses Among Yazidi Refugees by Age and Sex Prevalence among 67 Yazidi children aged 6 months to 11 years, 37 adolescents aged 11 to 17 years, and 126 adults (81 women and 45 men) 18 years and older.

The 10 most prevalent *ICD-10-CM *diagnostic codes by age group and sex are shown in Figure 2. Among 230 refugees, the most prevalent clinical diagnoses across all groups were abdominal and pelvic pain (47 patients [20.4%]), iron deficiency (43 patients [18.7%]), anemia (36 patients [15.7%]), and PTSD (33 patients [14.3%]). Among 67 Yazidi children, other prevalent diagnoses included acute upper respiratory infections (13 patients [19.4%]), enterobiasis (12 patients [17.9%]), short stature (8 patients [11.9%]), dental caries (8 patients [11.9%]), and eating disorders (7 patients [10.4%]). The most common diagnoses among 37 Yazidi adolescents were anemia (12 patients [32.4%]), visual disturbances (6 patients [16.2%]), unspecified hematuria (6 patients [16.2%]), and psychogenic nonepileptic seizures (PNES; <5 adolescents; 11 patients total [4.8% ]). Posttraumatic stress disorder was the most prevalent diagnosis among 81 women (25 patients [30.9%]), followed by nonorganic insomnia (17 patients [21.0%]) and low back pain (17 patients [21.0%]). The most prevalent diagnoses among 45 men were dyslipidemia (11 patients [24.4%]), dental caries (7 patients [15.6%]), joint pain (6 patients [13.3%]), and low back pain (6 patients [13.3%]). Posttraumatic stress disorder was not commonly diagnosed among children, adolescents, and men (<5 patients in each group) nor was depression (<5 patients in each group). All recorded diagnoses and frequencies by age group and sex are shown in eTables 2 to 6 in Supplement 1.

### Suspected Conditions Associated With Daesh Exposure According to Clinicians

**Figure 3.**
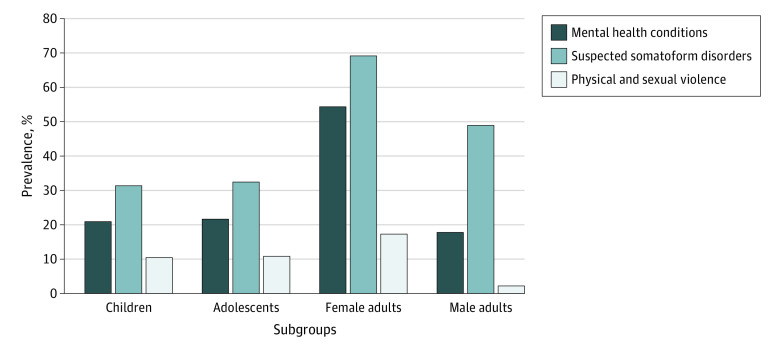
Prevalence of Suspected Daesh Exposure–Related Clinical Groups Among Yazidi Refugees by Age and Sex Prevalence among 67 children aged 6 months to 11 years, 37 adolescents aged 11 to 17 years, and 126 adults (81 women and 45 men) 18 years and older. The 3 clinical groups were identified by expert refugee clinicians as likely to be associated with Daesh exposure after a consensus-building process.

After reaching consensus, clinicians summarized diagnostic data into 3 clinical groups most likely to be associated with Daesh exposure: mental health conditions, suspected somatoform disorders, and conditions of sexual and physical violence. These clinical groupings combined diagnoses across different *ICD-10-CM *chapters otherwise not captured by the *ICD-10-CM *coding system alone. The diagnoses within each consensus-derived clinical group are shown in Table 2, and the prevalence of diagnoses in each clinical group by age group and sex is shown in Figure 3.

**Table 2.** Clinician Consensus-Derived Clinical Groups Likely Associated With Daesh Exposure Among Yazidi Refugees

Clinical group likely associated with Daesh exposure and *ICD-10-CM* code	Diagnosis	Participants, No. (%) (n = 230)^a^
Mental health conditions		
F43.1	Posttraumatic stress disorder	33 (14.3)
F51.0	Nonorganic insomnia	19 (8.3)
F32.9	Depressive episode, unspecified	13 (5.7)
F41	Other anxiety disorders	13 (5.7)
F44.5	Psychogenic nonepileptic seizures	11 (4.8)
F50.9	Eating disorder, unspecified	7 (3.0)
F43.8	Other reactions to severe stress	6 (2.6)
F41.0	Panic attacks	5 (2.2)
F51.5	Nightmares	5 (2.2)
F43.2	Adjustment disorder	CS
F43.0	Acute stress reaction	CS
R63.0	Anorexia	CS
F34.1	Dysthymia	CS
F38	Other mood disorders	CS
R45.8	Suicidal ideation	CS
F40.1	Social phobias	CS
Z60.9	Social isolation	CS
F91.9	Conduct disorders, unspecified	CS
Z91.5	Suicide attempt	CS
Suspected somatoform disorders		
R10	Abdominal and pelvic pain	47 (20.4)
M54.5	Low back pain	26 (11.3)
R51	Headaches	19 (8.3)
M25.5	Joint pain	19 (8.3)
G44.2	Tension-type headache	11 (4.8)
G43	Migraine	10 (4.3)
R42	Dizziness	9 (3.9)
N76.0	Acute vaginitis	8 (3.5)
L65	Hair loss	6 (2.6)
R52	Chronic pain	5 (2.2)
F98.0	Enuresis	CS
R47.8	Speech disturbances, unspecified	CS
N89.8	Vaginal discharge	CS
R07	Chest pain	CS
R53	Fatigue	CS
F80.9	Developmental disorder of speech and language, unspecified	CS
K58	Irritable bowel syndrome	CS
R20.8	Burning sensation	CS
Physical and sexual violence		
T74.2	Sexual abuse	8 (3.5)
Y05	Sexual assault by bodily force	6 (2.6)
T74.1	Physical abuse	5 (2.2)
Y07	Maltreatment (torture)	CS
S82.8	Fractures of lower leg	CS
Y36.2	Shrapnel injury	CS
S05.9	Injury of eye and orbit, unspecified	CS
S32.0	Fracture of lumbar vertebra	CS
S52.3	Fracture of radius	CS
T13.5	Injury of lower limb, unspecified	CS

Abbreviations: CS, cell suppression; *ICD-10-CM*, *International Statistical Classification of Diseases, Tenth Revision, Clinical Modification*.

^a^ Cells with fewer than 5 participants were suppressed to protect participant confidentiality.

Overall, 74 of 230 Yazidi refugees (32.2%) had diagnoses in the mental health conditions clinical group. For this clinical group, clinicians combined mental health diagnoses deemed to be associated with Daesh exposure, such as nightmares, panic attacks, eating disorders, and suicidal ideation (Table 2). More Yazidi women had diagnoses in this group (44 of 81 patients [54.3%]) compared with men (8 of 45 patients [17.8%]; *P* < .001), children (14 of 67 patients [20.9%]; *P* < .001), and adolescents (8 of 37 patients [21.6%]; *P* < .001). These values represent a 3-fold higher prevalence of mental health conditions likely associated with Daesh among women compared with men and children. The second consensus-derived clinical group combined physical conditions and symptoms that clinicians suspected were somatoform disorders but were not coded as such in patients’ records. Clinicians used their clinical expertise and existing literature on somatoform disorders among trauma survivors to derive suspected somatoform conditions, which included abdominal and pelvic pain, headaches, dizziness, hair loss, and vaginitis, among others (Table 2). Diagnoses in this clinical group were common among all Yazidi refugees (111 of 230 patients [48.3%]) but more frequent among women (56 of 81 patients [69.1%]) compared with men (22 of 45 patients [48.9%]; *P* = .03), children (21 of 67 patients [31.3%]; *P* < .001), and adolescents (12 of 37 patients [32.4%]; *P* < .001). The third consensus-derived clinical group included conditions of physical or sexual violence, such as sexual abuse, assault, physical abuse, and injuries (Table 2). Overall, 26 of 230 Yazidi refugees (11.3%) had diagnoses recorded in this clinical group, and these diagnoses occurred more frequently among women (14 of 81 patients [17.3%]) compared with men (<5 patients; *P* = .01).

## Discussion

In this cross-sectional study of Yazidi refugees who resettled in Canada, we found more than one-half (51.2%) of refugees had direct Daesh exposure and nearly all (95.2% of families) experienced family separations. Clinicians at a specialized refugee clinic recorded a high prevalence of mental and physical health conditions among these refugees. We also identified specific clinical groups of conditions that clinicians perceived were likely to be associated with Daesh exposure. To our knowledge, this study is the first to provide a comprehensive assessment of sociodemographic characteristics, health conditions, and family separations among resettled Yazidi refugees who survived the 2014 Daesh genocide.

Most resettled Yazidi refugees were vulnerable women and children who survived Daesh’s gendered violence and war crimes, as most Yazidi men were killed.^8^ Many refugees had structural vulnerabilities, such as limited English language proficiency, low literacy, and low educational attainment, which were especially pronounced among women who received little or no formal education.^8^ These vulnerabilities were compounded by the ongoing trauma of family separations, which collectively pose barriers to healthy resettlement and long-term recovery.^8,48,49^

Similar to previous studies,^8,50,51^ we identified common health conditions among newly resettled refugees, including infectious and parasitic diseases, chronic pain, and nutritional deficiencies. These health conditions reflected the poor living conditions Yazidis experienced in captivity and before resettlement, which included lack of sanitation, food, clean water, and/or health care. While malnutrition is common among refugees,^52^ we observed a higher prevalence of anemia among adolescents and a higher prevalence of dental caries among children compared with previous studies.^53,54,55^ The cohort of the current study had a higher proportion of women and girls, which may explain these findings; however, Yazidi refugees’ experiences of forced starvation, nutritional restriction, and violent treatment during captivity are likely also important factors.^8^ Our findings corroborate previous reports of poor oral health among refugees exposed to torture and trauma.^54,55^

Similar to studies of Yazidi refugees internally displaced in Iraq,^23,56,57^ we found mental and behavioral disorders were highly prevalent among resettled Yazidi refugees, especially women, who experienced a 3-fold higher prevalence of mental health conditions suspected to be associated with Daesh exposure compared with men. Previous studies^23,56,57^ have found that this high prevalence of mental health conditions among Yazidi female refugees was associated with the gender-based violence, sexual assault, and rape they experienced. However, we found lower rates of PTSD among female Yazidi refugees living in Canada (30.9%) than previously reported rates ranging from 60% to 80% among those living in IDP camps.^19,20,58^ Similarly, we found substantially lower rates of PTSD and depression compared with studies of displaced Yazidi children and adolescents in Iraq and Turkey.^21,25^ We also found fewer diagnoses of physical or sexual violence than expected considering the ubiquity of abuses recounted by Yazidi survivors.^8,9,48,59^ Notably, we found a high prevalence of PNES, which has been anecdotally associated with trauma among Yazidi refugees^59^ but is a rare condition among the general population.^60^ However, our observed rate of 4.8% was substantially lower than that of another study, which found PNES present in up to 44% of female Yazidi refugees.^61^

Together, these findings suggest potential underdiagnosis or underreporting of mental health conditions, such as PTSD, depression, PNES, and sexual trauma, at our clinic. These relatively low rates of mental health conditions may reflect the honeymoon phase, which describes a period of well-being that can occur early after resettlement.^62,63^ More likely, this discrepancy reflects the study site’s lack of routine mental health screening at intake, during which clinicians prefer to first establish trust with new patients. Furthermore, the limited length of stay at the time of medical record review and the substantial shortage of Kurmanji interpreters early after Yazidi resettlement^10^ likely prevented detailed psychosocial assessments and subsequent diagnosis of mental and behavioral disorders. In addition, previous studies^10,61,64^ have reported shame and stigma as barriers that prevented Yazidi refugees from sharing mental health concerns with clinicians.

Clinicians suspected nearly one-half (48.3%) of the physical symptoms diagnosed among Yazidi refugees represented somatoform disorders. These disorders, often underdiagnosed in EMRs, pose diagnostic challenges due to documentation and coding inconsistencies, potentially accounting for their initial absence in patient medical records.^36,37^ Previous research^27,28,29^ revealed that torture survivors and traumatized refugees resettled in Western countries often presented with more unexplained somatic symptoms than the general population. The physical symptoms clinicians identified as suspected somatoform disorders corroborate findings from a German study^31^ that reported pain, dizziness, paresthesia, gastrointestinal symptoms, and functional limitations as common somatic symptoms among female refugees traumatized by Daesh exposure. Other studies^28,29,32,65^ have also cited abdominal, joint, and genital pain as somatic symptoms among traumatized refugees and survivors of torture and sexual abuse. Many of the suspected somatoform disorders we highlighted have been previously associated with PTSD,^30^ and the high prevalence of somatoform disorders in the current cohort suggests that Yazidi refugees likely manifest their psychological trauma as physical symptoms.

The findings of this study suggest that Yazidi refugees require holistic trauma-informed care that considers the severe physical and psychological trauma they endured.^66^ Understanding cultural nuances, such as how family-oriented societies like the Yazidi community perceive and manifest trauma, is important to prevent misdiagnoses and ineffective treatments.^64,66^ Given that we collected EMR data shortly after refugees’ resettlement to Canada, we suspect that the health conditions described are more likely associated with acute preresettlement trauma and exposures rather than the postresettlement stressors often experienced by refugees.^67,68^ Thus, these findings may generalize to thousands of Yazidi refugees still internally displaced in Iraq.

Our findings may assist health care professionals and service organizations in better addressing the complex needs of Yazidi refugees as well as those of other genocide survivors. Regardless of whether individual Yazidi refugees were directly exposed to Daesh, all Yazidi refugees are genocide survivors who experience ongoing individual, collective, and transgenerational trauma^49^ and require special considerations of their cultural background and specialized health care needs. Our findings may also inform broader immigration and resettlement policies for future acutely traumatized refugees. These policies should aim to improve refugee community engagement, prioritize family reunifications, mitigate structural violence, and improve collaboration between health care and resettlement sectors. In addition, this study contributes to research and efforts to ensure that the genocide and human rights violations that Yazidi individuals experienced are documented and can perhaps help promote community healing.

### Limitations

This study has limitations. First, the study was conducted at a single site and included a relatively small sample. Despite this limitation, our site is within 1 of 4 Canadian jurisdictions to resettle Yazidi refugees, and our study identified 98.8% of the Yazidi refugees reported to have resettled locally, likely extending the generalizability of our findings.^8^ Second, our reliance on retrospective cross-sectional medical records review introduces potential sources of inaccuracies, including clinician underreporting, error, or missing results. Third, caution is necessary when interpreting our consensus-derived clinical groupings due to potential misclassification and bias. Nevertheless, our robust consensus process engaged clinician experts who provided direct patient care to Yazidi refugees during our study as well as Yazidi leaders with lived experience and nuanced cultural understanding, which collectively strengthen the validity of our findings. Despite these limitations, our study provides granular health information about newly resettled Yazidi refugees in Canada.

## Conclusions

This cross-sectional study found that Yazidi refugees who resettled in Canada after surviving the Daesh genocide experienced substantial trauma, complex mental and physical health conditions, and nearly universal family separations. These findings highlight the need for comprehensive health care, community engagement, and family reunification and may inform care for other refugees and genocide victims.

## Notes

Supplement 1. **eFigure.** Heat Map Analysis of *ICD-10-CM* Chapters Among Yazidi Refugees by Age and Sex **eTable 1.** Top 5 Most Prevalent *ICD-10-CM* Diagnoses by *ICD-10-CM* Chapter Among Yazidi Refugees (n = 230) **eTable 2.** Top 30 *ICD-10-CM* Diagnoses Among Yazidi Refugees (n = 230) **eTable 3.** Frequency of *ICD-10-CM* Diagnoses Among Children Aged 6 Months to 11 Years (n = 67) **eTable 4.** Frequency of *ICD-10-CM* Diagnoses Among Adolescents Aged 12 to 17 Years (n = 37) **eTable 5.** Frequency of *ICD-10-CM* Diagnoses Among Female Adults 18 Years or Older (n = 81) **eTable 6.** Frequency of *ICD-10-CM* Diagnoses Among Male Adults 18 Years or Older (n = 45)

Supplement 2.
Data Sharing Statement
