# Supplementary material for: Sociodemographic Characteristics and Mental and Physical Health Diagnoses of Yazidi Refugees Who Survived the Daesh Genocide and Resettled in Canada
Source: JAMA Netw Open. 2023 Jul 12;6(7):e2323064. doi: 10.1001/jamanetworkopen.2023.23064 (PMC10339151; doi:10.1001/jamanetworkopen.2023.23064)
Supplement: Supplement 1. — eFigure. Heat Map Analysis of ICD-10-CM Chapters Among Yazidi Refugees by Age and Sex eTable 1. Top 5 Most Prevalent ICD-10-CM Diagnoses by ICD-10-CM Chapter Among Yazidi Refugees (n = 230) eTable 2. Top 30 ICD-10-CM Diagnoses Among Yazidi Refugees (n = 230) eTable 3. Frequency of ICD-10-CM Diagnoses Among Children Aged 6 Months to 11 Years (n = 67) eTable 4. Frequency of ICD-10-CM Diagnoses Among Adolescents Aged 12 to 17 Years (n = 37) eTable 5. Frequency of ICD-10-CM Diagnoses Among Female Adults 18 Years or Older (n = 81) eTable 6. Frequency of ICD-10-CM Diagnoses Among Male Adults 18 Years or Older (n = 45) [file jamanetwopen-e2323064-s001.pdf]

Supplementary Online Content

Hassan N, Coakley A, Al Masri I, et al. Sociodemographic Characteristics and Mental and Physical Health Diagnoses of Yazidi Refugees Who Survived the Daesh Genocide and Resettled in Canada. *JAMA Netw Open*. 2023;6(7):e2323064. doi:10.1001/jamanetworkopen.2023.23064

eFigure. Heat Map Analysis of *ICD-10-CM* Chapters Among Yazidi Refugees by Age and Sex

eTable 1. Top 5 Most Prevalent *ICD-10-CM* Diagnoses by *ICD-10-CM* Chapter Among Yazidi Refugees (n = 230)

eTable 2. Top 30 *ICD-10-CM* Diagnoses Among Yazidi Refugees (n = 230)

eTable 3. Frequency of *ICD-10-CM* Diagnoses Among Children Aged 6 Months to 11 Years (n = 67)

eTable 4. Frequency of *ICD-10-CM* Diagnoses Among Adolescents Aged 12 to 17 Years (n = 37)

eTable 5. Frequency of *ICD-10-CM* Diagnoses Among Female Adults 18 Years or Older (n = 81)

eTable 6. Frequency of *ICD-10-CM* Diagnoses Among Male Adults 18 Years or Older (n = 45)

This supplemental material has been provided by the authors to give readers additional information about their work.

© 2023 Hassan N et al. *JAMA Network Open*.

eFigure. Heat Map Analysis of *ICD-10-CM* Chapters Among Yazidi Refugees by Age and Sex

ICD-10-CM Chapter		Description	Overall (n=230)	Children (n = 67)	Adolescent (n = 37)	Female Adults (n = 81)	Male Adults (n = 45)
**18**	R00–R99	Symptoms, signs and abnormal clinical and laboratory findings, not elsewhere classified	49.1	43.3	37.8	58.0	51.1
**4**	E00–E90	Endocrine, nutritional and metabolic diseases	37.4	40.3	37.8	37.0	33.3
**5**	F00–F99	Mental and behavioural disorders	33.5	25.4	24.3	54.3	15.6
**1**	A00–B99	Certain infectious and parasitic diseases	31.3	38.8	21.6	30.9	28.9
**13**	M00–M99	Diseases of the musculoskeletal system and connective tissue	28.7	9.0	21.6	40.7	42.2
**11**	K00–K93	Diseases of the digestive system	27.0	16.4	2.7	37.0	44.4
**14**	N00–N99	Diseases of the genitourinary system	21.7	9.0	10.8	43.2	11.1
**7**	H00–H59	Diseases of the eye and adnexa	21.7	9.0	29.7	25.9	26.7
**3**	D50–D89	Diseases of the blood and blood-forming organs and certain disorders involving the immune mechanism	21.3	17.9	35.1	24.7	8.9
**12**	L00–L99	Diseases of the skin and subcutaneous tissue	20.0	11.9	16.2	27.2	22.2
**10**	J00–J99	Diseases of the respiratory system	15.7	23.9	2.7	11.1	22.2
**19**	S00–T98	Injury, poisoning, and certain other consequences of external causes	11.7	11.9	13.5	14.8	4.4
**6**	G00–G99	Diseases of the nervous system	10.4	0.0	5.4	23.5	6.7
**8**	H60–H95	Diseases of the ear and mastoid process	9.6	6.0	2.7	12.3	15.6
**9**	I00–I99	Diseases of the circulatory system	6.1	0.0	0.0	12.3	8.9
**2**	C00–D48	Neoplasms	5.2	9.0	0.0	6.2	2.2
**20**	V01–Y98	External causes of morbidity and mortality	4.8	3.0	2.7	9.9	0.0
**21**	Z00–Z99	Factors influencing health status and contact with health services	2.6	0.0	0.0	6.2	2.2
**17**	Q00–Q99	Congenital malformations, deformations, and chromosomal abnormalities	2.2	6.0	0.0	1.2	0.0
**15**	O00–O99	Pregnancy, childbirth, and the puerperium	1.3	0.0	0.0	3.7	0.0

Prevalence of diagnostic chapter (% of diagnoses in each age group)

≥50 | 40 | 30 | 20 | 10 | 0

© 2023 Hassan N et al. *JAMA Network Open*.

eTable 1. Top 5 Most Prevalent *ICD-10-CM* Diagnoses by *ICD-10-CM* Chapter Among Yazidi Refugees (n = 230)

Chapters are ordered from most to least prevalent. Not shown are *ICD-10-CM* chapters prevalent in fewer than 10% of the entire cohort.

ICD-10-CM chapter	ICD-10 code	ICD-10 description	n	%
R00–R99: Symptoms, signs and abnormal clinical findings	R10	Abdominal and pelvic pain	47	20.4
	R51	Headache	19	8.3
	R31	Unspecified haematuria	18	7.8
	R30.0	Dysuria	11	4.8
	R21	Rash	9	3.9
E00–E90: Endocrine, nutritional and metabolic diseases	E61.1	Iron deficiency	43	18.7
	E78	Dyslipidemia	24	10.4
	E34.3	Short stature, not elsewhere classified	10	4.3
	E53.8	Deficiency of other specified B group vitamins	7	3.0
	E66	Obesity	6	2.6
F00–F99: Mental and behavioural disorders	F43.1	Post-traumatic stress disorder	33	14.3
	F51.0	Nonorganic insomnia	19	8.3
	F32.9	Depression	13	5.7
	F41	Other anxiety disorders	13	5.7
	F44.5	Psychogenic non-epileptic seizures	11	4.8
A00–B99: Infectious and parasitic diseases	B80	Enterobiasis	19	8.3
	B98.0	Helicobacter pylori [H.pylori]	15	6.5
	A07.1	Giardia	9	3.9
	A15.9	Respiratory tuberculosis unspecified	9	3.9
	B65	Schistosomiasis [bilharziasis]	9	3.9
M00–M99: Musculoskeletal conditions	M54.5	Low back pain	26	11.3
	M25.5	Pain in joint	19	8.3
	M79.6	Pain in leg	13	5.7
	M62.6	Muscle strain	6	2.6
	M21.9	Acquired deformity of limb, unspecified	CS	CS
K00–K93: Diseases of the digestive system	K02	Dental caries	24	10.4
	K59.0	Constipation	14	6.1
	K64	Hemorrhoids	10	4.3
	K08.8	Dental pain	9	3.9
	K29.7	Gastritis	6	2.6
	N39.0	Urinary tract infection, site not specified	10	4.3
	N76.0	Acute vaginitis	8	3.5

© 2023 Hassan N et al. *JAMA Network Open*.

N00–N99: Gynecologic conditions	N94.6	Dysmenorrhoea, unspecified	7	3
	N39.4	Other specified urinary incontinence	6	2.6
	N92.0	Menorrhagia	CS	CS
H00–H59: Conditions of vision	H53	Visual disturbances	22	9.6
	H52	Disorders of refraction and accommodation	14	6.1
	H26	Other cataract	CS	CS
	H11.0	Pterygium	CS	CS
	H52.2	Astigmatism	CS	CS
D50–D89: Blood conditions	D50	Iron deficiency anaemia	36	15.7
	D47.3	Thrombocytosis	8	3.5
	D72.1	Eosinophilia	5	2.2
	D56.3	Thalassaemia trait	CS	CS
	D64	Other anaemias	CS	CS
L00–L99: Skin conditions	L70	Acne	14	6.1
	L30.9	Dermatitis, unspecified	8	3.5
	L65	Hair loss	6	2.6
	L25	Unspecified contact dermatitis	CS	CS
	L29	Pruritus	CS	CS
J00–J99: Respiratory conditions	J06	Upper Respiratory Tract Infection	26	11.3
	J34.8	Other specified disorders of nose and nasal sinuses	CS	CS
	J02	Acute pharyngitis	CS	CS
	J01	Acute sinusitis	CS	CS
	J02.0	Streptococcal pharyngitis	CS	CS
S00–T98: Injury and poisoning	T74.2	Sexual abuse	8	3.5
	T74.1	Physical abuse	5	2.2
	S61	Open wound of wrist and hand	CS	CS
	S62.8	Fracture of wrist	CS	CS
	S82.8	Fracture of ankle	CS	CS
G00–G99: Diseases of the nervous system	G44.2	Tension-type headache	11	4.8
	G43	Migraine	10	4.3
	G62.9	Polyneuropathy, unspecified	CS	CS
	G47.9	Sleep disorder, unspecified	CS	CS
	G82.2	Paraplegia, unspecified	CS	CS

Abbreviation: CS, Cell Suppression. Cells with fewer than 5 participants were suppressed to protect participant confidentiality.

© 2023 Hassan N et al. *JAMA Network Open*.

eTable 2. Top 30 *ICD-10-CM* Diagnoses Among Yazidi Refugees (n = 230)

ICD-10-CM code	Description	n	%
R10	Abdominal and pelvic pain	47	20.4
E61.1	Iron deficiency	43	18.7
D50	Iron deficiency anaemia	36	15.7
F43.1	Post-traumatic stress disorder	33	14.3
J06	Acute upper respiratory infections of multiple and unspecified sites	26	11.3
M54.5	Low back pain	26	11.3
E78	Disorders of lipoprotein metabolism and other lipidaemias	24	10.4
K02	Dental caries	24	10.4
H53	Visual disturbances	22	9.6
B80	Enterobiasis	19	8.3
F51.0	Nonorganic insomnia	19	8.3
M25.5	Pain in joint	19	8.3
R51	Headache	19	8.3
R31	Unspecified haematuria	18	7.8
B98.0	Helicobacter pylori [H.pylori] as the cause of diseases classified to other chapters	15	6.5
H52	Disorders of refraction and accommodation	14	6.1
K59.0	Constipation	14	6.1
L70	Acne	14	6.1
F32.9	Depressive episode, unspecified	13	5.7
F41	Other anxiety disorders	13	5.7
H91	Other hearing loss	13	5.7
M79.6	Pain in limb	13	5.7
I10	Essential (primary) hypertension	12	5.2
F44.5	Psychogenic non-epileptic seizures	11	4.8
G44.2	Tension-type headache	11	4.8
R30.0	Dysuria	11	4.8
E34.3	Short stature, not elsewhere classified	10	4.3
G43	Migraine	10	4.3
K64	Haemorrhoids and perianal venous thrombosis	10	4.3
N39.0	Urinary tract infection, site not specified	10	4.3

© 2023 Hassan N et al. *JAMA Network Open*.

eTable 3. Frequency of *ICD-10-CM* Diagnoses Among Children Aged 6 Months to 11 Years (n = 67)

ICD-10-CM code	Description	n	%
E61.1	Iron deficiency	19	28.4
J06	Acute upper respiratory infections of multiple and unspecified sites	13	19.4
R10	Abdominal and pelvic pain	13	19.4
B80	Enterobiasis	12	17.9
E34.3	Short stature, not elsewhere classified	8	11.9
K02	Dental caries	8	11.9
D50	Iron deficiency anaemia	7	10.4
F50.9	Eating disorder, unspecified	7	10.4
A07.1	Giardiasis [lambliasis]	6	9.0
R31	Unspecified haematuria	6	9.0
A07.8	Other specified protozoal intestinal diseases	5	7.5
D47.3	Essential (haemorrhagic) thrombocythaemia	5	7.5
L30.9	Dermatitis, unspecified	5	7.5
R30.0	Dysuria	5	7.5
B07	Viral warts	CS	CS
F43.1	Post-traumatic stress disorder	CS	CS
K59.0	Constipation	CS	CS
T74.1	Physical abuse	CS	CS
B65	Schistosomiasis [bilharziasis]	CS	CS
B71.0	Hymenolepiasis	CS	CS
D72.1	Eosinophilia	CS	CS
F98.0	Nonorganic enuresis	CS	CS
H52	Disorders of refraction and accommodation	CS	CS
N39.4	Other specified urinary incontinence	CS	CS
R05	Cough	CS	CS
R47.8	Other and unspecified speech disturbances	CS	CS
B98.0	Helicobacter pylori [H.pylori] as the cause of diseases classified to other chapters	CS	CS
D55.0	Anaemia due to glucose-6-phosphate dehydrogenase [G6PD] deficiency	CS	CS
F80.9	Developmental disorder of speech and language, unspecified	CS	CS
H91	Other hearing loss	CS	CS
M25.5	Pain in joint	CS	CS
N39.0	Urinary tract infection, site not specified	CS	CS
R01.1	Cardiac murmur, unspecified	CS	CS

© 2023 Hassan N et al. *JAMA Network Open*.

R32	Unspecified urinary incontinence	CS	CS
R51	Headache	CS	CS
R62.8	Other lack of expected normal physiological development	CS	CS
A15.9	Respiratory tuberculosis unspecified, confirmed bacteriologically and histologically	CS	CS
B89	Unspecified parasitic disease	CS	CS
D48.5	Neoplasm of uncertain or unknown behaviour: Skin	CS	CS
D70	Agranulocytosis	CS	CS
E03.9	Hypothyroidism, unspecified	CS	CS
E53.8	Deficiency of other specified B group vitamins	CS	CS
E66	Obesity	CS	CS
F41	Other anxiety disorders	CS	CS
F43.2	Adjustment disorders	CS	CS
F43.8	Other reactions to severe stress	CS	CS
F82	Specific developmental disorder of motor function	CS	CS
F91.9	Conduct disorder, unspecified	CS	CS
H02.4	Ptosis of eyelid	CS	CS
H50	Other strabismus	CS	CS
H53	Visual disturbances	CS	CS
H53.2	Diplopia	CS	CS
H61.2	Impacted cerumen	CS	CS
H91.3	Deaf mutism, not elsewhere classified	CS	CS
H92.0	Otalgia	CS	CS
J02.0	Streptococcal pharyngitis	CS	CS
J18.9	Pneumonia, unspecified	CS	CS
J31	Chronic rhinitis, nasopharyngitis and pharyngitis	CS	CS
J34.8	Other specified disorders of nose and nasal sinuses	CS	CS
J35.1	Hypertrophy of tonsils	CS	CS
K04.7	Periapical abscess without sinus	CS	CS
L05.9	Pilonidal cyst without abscess	CS	CS
L21	Seborrhoeic dermatitis	CS	CS
L22	Diaper [napkin] dermatitis	CS	CS
L65	Other nonscarring hair loss	CS	CS
M40.2	Other and unspecified kyphosis	CS	CS
M43.6	Torticollis	CS	CS
M54.5	Low back pain	CS	CS
M79.6	Pain in limb	CS	CS
N13.7	Vesicoureteral-reflux-associated uropathy	CS	CS

© 2023 Hassan N et al. *JAMA Network Open*.

N76.0	Acute vaginitis	CS	CS
Q53.1	Undescended testicle, unilateral	CS	CS
Q55.2	Other congenital malformations of testis and scrotum	CS	CS
Q60.0	Renal agenesis, unilateral	CS	CS
Q68.8	Other specified congenital musculoskeletal deformities	CS	CS
R04.0	Epistaxis	CS	CS
R11	Nausea and vomiting	CS	CS
R21	Rash and other nonspecific skin eruption	CS	CS
R26.8	Other and unspecified abnormalities of gait and mobility	CS	CS
R27.8	Other and unspecified lack of coordination	CS	CS
R35	Polyuria	CS	CS
R39.1	Other difficulties with micturition	CS	CS
R42	Dizziness and giddiness	CS	CS
R63.1	Polydipsia	CS	CS
R80	Isolated proteinuria	CS	CS
S05.9	Injury of eye and orbit, unspecified	CS	CS
S52.3	Fracture of shaft of radius	CS	CS
S61	Open wound of wrist and hand	CS	CS
S62.8	Fracture of other and unspecified parts of wrist and hand	CS	CS
S91.2	Open wound of toe(s) with damage to nail	CS	CS
Y05	Sexual assault by bodily force	CS	CS
Y36.2	War operations involving other explosions and fragments	CS	CS
**Total**		235	100

Abbreviation: CS, Cell Suppression. Cells with fewer than 5 participants were suppressed to protect participant confidentiality.

© 2023 Hassan N et al. *JAMA Network Open*.

eTable 4. Frequency of *ICD-10-CM* Diagnoses Among Adolescents Aged 12 to 17 Years (n = 37)

ICD-10-CM code	Description	n	%
D50	Iron deficiency anaemia	12	32.4
E61.1	Iron deficiency	11	29.7
H53	Visual disturbances	6	16.2
R31	Unspecified haematuria	6	16.2
R10	Abdominal and pelvic pain	5	13.5
F44.5	Psychogenic non-epileptic seizures	CS	CS
L70	Acne	CS	CS
N94.6	Dysmenorrhoea, unspecified	CS	CS
B80	Enterobiasis	CS	CS
F43.1	Post-traumatic stress disorder	CS	CS
H52	Disorders of refraction and accommodation	CS	CS
M21.9	Acquired deformity of limb, unspecified	CS	CS
R51	Headache	CS	CS
B65	Schistosomiasis [bilharziasis]	CS	CS
D70	Agranulocytosis	CS	CS
E53.8	Deficiency of other specified B group vitamins	CS	CS
F51.0	Nonorganic insomnia	CS	CS
G43	Migraine	CS	CS
M25.5	Pain in joint	CS	CS
M54.5	Low back pain	CS	CS
M79.6	Pain in limb	CS	CS
T74.2	Sexual abuse	CS	CS
A07.1	Giardiasis [lambliasis]	CS	CS
B00	Herpesviral [herpes simplex] infections	CS	CS
B07	Viral warts	CS	CS
B85.0	Pediculosis due to Pediculus humanus capitis	CS	CS
B98.0	Helicobacter pylori [H.pylori] as the cause of diseases classified to other chapters	CS	CS
D72.1	Eosinophilia	CS	CS
E34.3	Short stature, not elsewhere classified	CS	CS
F43.2	Adjustment disorders	CS	CS
F51.3	Sleepwalking [somnambulism]	CS	CS
F51.4	Sleep terrors [night terrors]	CS	CS
F51.5	Nightmares	CS	CS
H43.3	Other vitreous opacities	CS	CS

© 2023 Hassan N et al. *JAMA Network Open*.

H54	Visual impairment including blindness (binocular or monocular)	CS	CS
H91.3	Deaf mutism, not elsewhere classified	CS	CS
J06	Acute upper respiratory infections of multiple and unspecified sites	CS	CS
K02	Dental caries	CS	CS
L21	Seborrhoeic dermatitis	CS	CS
L85.3	Xerosis cutis	CS	CS
M62.6	Muscle strain	CS	CS
N76.0	Acute vaginitis	CS	CS
R01.1	Cardiac murmur, unspecified	CS	CS
R05	Cough	CS	CS
R25.2	Cramp and spasm	CS	CS
R42	Dizziness and giddiness	CS	CS
R80	Isolated proteinuria	CS	CS
S06.0	Concussion	CS	CS
S60.0	Contusion of finger(s) without damage to nail	CS	CS
T13.5	Injury of unspecified muscle and tendon of lower limb, level unspecified	CS	CS
Y05	Sexual assault by bodily force	CS	CS
**Total**		114	100

Abbreviation: CS, Cell Suppression. Cells with fewer than 5 participants were suppressed to protect participant confidentiality.

© 2023 Hassan N et al. *JAMA Network Open*.

eTable 5. Frequency of *ICD-10-CM* Diagnoses Among Female Adults 18 Years or Older (n = 81)

ICD-10-CM code	Description	n	%
F43.1	Post-traumatic stress disorder	25	30.9
R10	Abdominal and pelvic pain	21	25.9
F51.0	Nonorganic insomnia	17	21.0
M54.5	Low back pain	17	21.0
D50	Iron deficiency anaemia	16	19.8
E78	Disorders of lipoprotein metabolism and other lipidaemias	13	16.0
R51	Headache	13	16.0
F32.9	Depressive episode, unspecified	12	14.8
E61.1	Iron deficiency	11	13.6
F41	Other anxiety disorders	10	12.3
H53	Visual disturbances	10	12.3
G44.2	Tension-type headache	9	11.1
L70	Acne	9	11.1
M25.5	Pain in joint	9	11.1
B98.0	Helicobacter pylori [H.pylori] as the cause of diseases classified to other chapters	8	9.9
G43	Migraine	8	9.9
I10	Essential (primary) hypertension	8	9.9
K02	Dental caries	8	9.9
N39.0	Urinary tract infection, site not specified	8	9.9
F44.5	Psychogenic non-epileptic seizures	7	8.6
J06	Acute upper respiratory infections of multiple and unspecified sites	7	8.6
M79.6	Pain in limb	7	8.6
H91	Other hearing loss	6	7.4
N76.0	Acute vaginitis	6	7.4
T74.2	Sexual abuse	6	7.4
A15.9	Respiratory tuberculosis unspecified, confirmed bacteriologically and histologically	5	6.2
E11	Type 2 diabetes mellitus	5	6.2
F41.0	Panic disorder [episodic paroxysmal anxiety]	5	6.2
H52	Disorders of refraction and accommodation	5	6.2
K59.0	Constipation	5	6.2
K64	Haemorrhoids and perianal venous thrombosis	5	6.2
R30.0	Dysuria	5	6.2
R31	Unspecified haematuria	5	6.2
R42	Dizziness and giddiness	5	6.2

B37.3	Candidiasis of vulva and vagina	CS	CS
F51.5	Nightmares	CS	CS
K08.8	Other specified disorders of teeth and supporting structures	CS	CS
K29.7	Gastritis, unspecified	CS	CS
L65	Other nonscarring hair loss	CS	CS
M62.6	Muscle strain	CS	CS
N92.0	Excessive and frequent menstruation with regular cycle	CS	CS
R06.0	Dyspnoea	CS	CS
Y05	Sexual assault by bodily force	CS	CS
Y07	Other maltreatment	CS	CS
B80	Enterobiasis	CS	CS
D47.3	Essential (haemorrhagic) thrombocythaemia	CS	CS
D56.3	Thalassaemia trait	CS	CS
D64	Other anaemias	CS	CS
E03.9	Hypothyroidism, unspecified	CS	CS
E53.8	Deficiency of other specified B group vitamins	CS	CS
E66	Obesity	CS	CS
F43.8	Other reactions to severe stress	CS	CS
G62.9	Polyneuropathy, unspecified	CS	CS
H26	Other cataract	CS	CS
I51.7	Cardiomegaly	CS	CS
K04.7	Periapical abscess without sinus	CS	CS
M54.3	Sciatica	CS	CS
N89.8	Other specified noninflammatory disorders of vagina	CS	CS
N92.6	Irregular menstruation, unspecified	CS	CS
N94.6	Dysmenorrhoea, unspecified	CS	CS
O03	Spontaneous abortion	CS	CS
R05	Cough	CS	CS
R11	Nausea and vomiting	CS	CS
R21	Rash and other nonspecific skin eruption	CS	CS
A09.0	Other and unspecified gastroenteritis and colitis of infectious origin	CS	CS
B65	Schistosomiasis [bilharziasis]	CS	CS
D69.6	Thrombocytopenia, unspecified	CS	CS
F43.2	Adjustment disorders	CS	CS
H11.0	Pterygium	CS	CS
H52.2	Astigmatism	CS	CS
H54	Visual impairment including blindness (binocular or monocular)	CS	CS

© 2023 Hassan N et al. *JAMA Network Open*.

H61.2	Impacted cerumen	CS	CS
I25	Chronic ischaemic heart disease	CS	CS
K04.9	Other and unspecified diseases of pulp and periapical tissues	CS	CS
K13.7	Other and unspecified lesions of oral mucosa	CS	CS
K21	Gastro-oesophageal reflux disease	CS	CS
K30	Functional dyspepsia	CS	CS
K76.0	Fatty (change of) liver, not elsewhere classified	CS	CS
L25	Unspecified contact dermatitis	CS	CS
L29	Pruritus	CS	CS
M19	Other arthrosis	CS	CS
M41.9	Scoliosis, unspecified	CS	CS
M51.3	Other specified intervertebral disc degeneration	CS	CS
N18	Chronic kidney disease	CS	CS
N20.0	Calculus of kidney	CS	CS
N28.1	Cyst of kidney, acquired	CS	CS
N30.9	Cystitis, unspecified	CS	CS
N39.4	Other specified urinary incontinence	CS	CS
N63	Unspecified lump in breast	CS	CS
N64.4	Mastodynia	CS	CS
N83.2	Other and unspecified ovarian cysts	CS	CS
N91.2	Amenorrhoea, unspecified	CS	CS
N97	Female infertility	CS	CS
R07	Pain in throat and chest	CS	CS
R19.6	Halitosis	CS	CS
R22.2	Localized swelling, mass and lump, trunk	CS	CS
R52	Pain, not elsewhere classified	CS	CS
R53	Malaise and fatigue	CS	CS
Z34.9	Supervision of normal pregnancy, unspecified	CS	CS
A04.7	Enterocolitis due to Clostridium difficile	CS	CS
A07.1	Giardiasis [lambliasis]	CS	CS
A07.8	Other specified protozoal intestinal diseases	CS	CS
B07	Viral warts	CS	CS
B36.9	Superficial mycosis, unspecified	CS	CS
B64	Unspecified protozoal disease	CS	CS
C06.0	Cheek mucosa	CS	CS
D25.9	Leiomyoma of uterus, unspecified	CS	CS
D68.9	Coagulation defect, unspecified	CS	CS
D72.1	Eosinophilia	CS	CS

© 2023 Hassan N et al. *JAMA Network Open*.

E04	Other nontoxic goitre	CS	CS
E86	Volume depletion	CS	CS
E87.5	Hyperkalaemia	CS	CS
F34.1	Dysthymia	CS	CS
F38	Other mood [affective] disorders	CS	CS
F40.1	Social phobias	CS	CS
F43.0	Acute stress reaction	CS	CS
G47.9	Sleep disorder, unspecified	CS	CS
G82.2	Paraplegia, unspecified	CS	CS
H02.9	Disorder of eyelid, unspecified	CS	CS
H10.1	Acute atopic conjunctivitis	CS	CS
H17	Corneal scars and opacities	CS	CS
H35.3	Degeneration of macula and posterior pole	CS	CS
H52.1	Myopia	CS	CS
H57.1	Ocular pain	CS	CS
H60.9	Otitis externa, unspecified	CS	CS
H66.9	Otitis media, unspecified	CS	CS
H72	Perforation of tympanic membrane	CS	CS
I21.9	Acute myocardial infarction, unspecified	CS	CS
I50.0	Congestive heart failure	CS	CS
I50.1	Left ventricular failure	CS	CS
I95.1	Orthostatic hypotension	CS	CS
J01	Acute sinusitis	CS	CS
J02	Acute pharyngitis	CS	CS
J30.4	Allergic rhinitis, unspecified	CS	CS
J31.0	Chronic rhinitis	CS	CS
K12.0	Recurrent oral aphthae	CS	CS
K35	Acute appendicitis	CS	CS
K58	Irritable bowel syndrome	CS	CS
K60.2	Anal fissure, unspecified	CS	CS
K62.5	Haemorrhage of anus and rectum	CS	CS
K80	Cholelithiasis	CS	CS
L02.2	Cutaneous abscess, furuncle and carbuncle of trunk	CS	CS
L28.0	Lichen simplex chronicus	CS	CS
L30.9	Dermatitis, unspecified	CS	CS
L68.0	Hirsutism	CS	CS
L73.9	Follicular disorder, unspecified	CS	CS
L80	Vitiligo	CS	CS
L81.1	Chloasma	CS	CS

© 2023 Hassan N et al. *JAMA Network Open*.

L81.9	Disorder of pigmentation, unspecified	CS	CS
L85.3	Xerosis cutis	CS	CS
M13.9	Arthritis, unspecified	CS	CS
M50.3	Other cervical disc degeneration	CS	CS
M67.4	Ganglion	CS	CS
M70.4	Prepatellar bursitis	CS	CS
M72.2	Plantar fascial fibromatosis	CS	CS
M75	Shoulder lesions	CS	CS
M75.1	Rotator cuff syndrome	CS	CS
M79.1	Myalgia	CS	CS
M81.9	Osteoporosis, unspecified	CS	CS
N08.3	Glomerular disorders in diabetes mellitus	CS	CS
N10	Acute tubulo-interstitial nephritis	CS	CS
N13.3	Other and unspecified hydronephrosis	CS	CS
N46	Male infertility	CS	CS
N60	Benign mammary dysplasia	CS	CS
N71	Inflammatory disease of uterus, except cervix	CS	CS
N72	Inflammatory disease of cervix uteri	CS	CS
N73.9	Female pelvic inflammatory disease, unspecified	CS	CS
N80.0	Endometriosis of uterus	CS	CS
N85.0	Endometrial glandular hyperplasia	CS	CS
N86	Erosion and ectropion of cervix uteri	CS	CS
N91.1	Secondary amenorrhoea	CS	CS
N92.1	Excessive and frequent menstruation with irregular cycle	CS	CS
N96	Habitual aborter	CS	CS
N97.9	Female infertility, unspecified	CS	CS
Q51.9	Congenital malformation of uterus and cervix, unspecified	CS	CS
R04.0	Epistaxis	CS	CS
R13	Dysphagia	CS	CS
R14	Flatulence and related conditions	CS	CS
R17	Unspecified jaundice	CS	CS
R20.8	Other and unspecified disturbances of skin sensation	CS	CS
R22.0	Localized swelling, mass and lump, head	CS	CS
R22.3	Localized swelling, mass and lump, upper limb	CS	CS
R22.4	Localized swelling, mass and lump, lower limb	CS	CS
R22.4	Localized swelling, mass and lump, lower limb	CS	CS
R25.2	Cramp and spasm	CS	CS
R26.1	Paralytic gait	CS	CS
R41.3	Other amnesia	CS	CS

© 2023 Hassan N et al. *JAMA Network Open*.

R45.8	Other symptoms and signs involving emotional state	CS	CS
R63.4	Abnormal weight loss	CS	CS
S61	Open wound of wrist and hand	CS	CS
S62.8	Fracture of other and unspecified parts of wrist and hand	CS	CS
S63.5	Sprain and strain of wrist	CS	CS
S82.8	Fractures of other parts of lower leg	CS	CS
T74.1	Physical abuse	CS	CS
T75.3	Motion sickness	CS	CS
T78.4	Allergy, unspecified	CS	CS
Z74	Problems related to care-provider dependency	CS	CS
Z90.4	Acquired absence of other parts of digestive tract	CS	CS
Z91.5	Personal history of self-harm	CS	CS
**Total**		586	100

Abbreviation: CS, Cell Suppression. Cells with fewer than 5 participants were suppressed to protect participant confidentiality.

© 2023 Hassan N et al. *JAMA Network Open*.

eTable 6. Frequency of *ICD-10-CM* Diagnoses Among Male Adults 18 Years or Older (n = 45)

ICD-10-CM code	Description	n	%
E78	Disorders of lipoprotein metabolism and other lipidaemias	11	24.4
R10	Abdominal and pelvic pain	8	17.8
K02	Dental caries	7	15.6
M25.5	Pain in joint	6	13.3
M54.5	Low back pain	6	13.3
H53	Visual disturbances	5	11.1
H91	Other hearing loss	5	11.1
J06	Acute upper respiratory infections of multiple and unspecified sites	5	11.1
K08.8	Other specified disorders of teeth and supporting structures	5	11.1
K59.0	Constipation	5	11.1
K64	Haemorrhoids and perianal venous thrombosis	5	11.1
R21	Rash and other nonspecific skin eruption	5	11.1
B98.0	Helicobacter pylori [H.pylori] as the cause of diseases classified to other chapters	CS	CS
I10	Essential (primary) hypertension	CS	CS
A15.9	Respiratory tuberculosis unspecified, confirmed bacteriologically and histologically	CS	CS
H52	Disorders of refraction and accommodation	CS	CS
K21	Gastro-oesophageal reflux disease	CS	CS
M79.6	Pain in limb	CS	CS
R52	Pain, not elsewhere classified	CS	CS
A07.8	Other specified protozoal intestinal diseases	CS	CS
B65	Schistosomiasis [bilharziasis]	CS	CS
E61.1	Iron deficiency	CS	CS
E66	Obesity	CS	CS
F41	Other anxiety disorders	CS	CS
F43.8	Other reactions to severe stress	CS	CS
G44.2	Tension-type headache	CS	CS
H10	Conjunctivitis	CS	CS
H92.0	Otalgia	CS	CS
J34.8	Other specified disorders of nose and nasal sinuses	CS	CS
K29.7	Gastritis, unspecified	CS	CS
L25	Unspecified contact dermatitis	CS	CS
L30.9	Dermatitis, unspecified	CS	CS
L90.5	Scar conditions and fibrosis of skin	CS	CS

© 2023 Hassan N et al. *JAMA Network Open*.

M51.1	Lumbar and other intervertebral disc disorders with radiculopathy	CS	CS
N46	Male infertility	CS	CS
R42	Dizziness and giddiness	CS	CS
A07.1	Giardiasis [lambliasis]	CS	CS
A09.0	Other and unspecified gastroenteritis and colitis of infectious origin	CS	CS
A63.0	Anogenital (venereal) warts	CS	CS
B35.3	Tinea pedis	CS	CS
B80	Enterobiasis	CS	CS
B86	Scabies	CS	CS
D48.5	Neoplasm of uncertain or unknown behaviour: Skin	CS	CS
D50	Iron deficiency anaemia	CS	CS
D56.3	Thalassaemia trait	CS	CS
D69.6	Thrombocytopenia, unspecified	CS	CS
D72.8	Other specified disorders of white blood cells	CS	CS
E34.3	Short stature, not elsewhere classified	CS	CS
E53.8	Deficiency of other specified B group vitamins	CS	CS
F32.9	Depressive episode, unspecified	CS	CS
F43.0	Acute stress reaction	CS	CS
F43.1	Post-traumatic stress disorder	CS	CS
G62.9	Polyneuropathy, unspecified	CS	CS
H11.0	Pterygium	CS	CS
H26	Other cataract	CS	CS
H44.2	Degenerative myopia	CS	CS
H52.1	Myopia	CS	CS
H52.2	Astigmatism	CS	CS
H91.3	Deaf mutism, not elsewhere classified	CS	CS
I25	Chronic ischaemic heart disease	CS	CS
J02	Acute pharyngitis	CS	CS
J02.9	Acute pharyngitis, unspecified	CS	CS
J44	Other chronic obstructive pulmonary disease	CS	CS
J45	Asthma	CS	CS
K07.6	Temporomandibular joint disorders	CS	CS
K30	Functional dyspepsia	CS	CS
K35	Acute appendicitis	CS	CS
K60.2	Anal fissure, unspecified	CS	CS
K76.0	Fatty (change of) liver, not elsewhere classified	CS	CS
K76.9	Liver disease, unspecified	CS	CS
L29	Pruritus	CS	CS

© 2023 Hassan N et al. *JAMA Network Open*.

L65	Other nonscarring hair loss	CS	CS
L70	Acne	CS	CS
L80	Vitiligo	CS	CS
L85.3	Xerosis cutis	CS	CS
M13.9	Arthritis, unspecified	CS	CS
M22.4	Chondromalacia patellae	CS	CS
M54.6	Pain in thoracic spine	CS	CS
M62.6	Muscle strain	CS	CS
M72.2	Plantar fascial fibromatosis	CS	CS
M75.1	Rotator cuff syndrome	CS	CS
M79.2	Neuralgia and neuritis, unspecified	CS	CS
M81.9	Osteoporosis, unspecified	CS	CS
N20.0	Calculus of kidney	CS	CS
N39.4	Other specified urinary incontinence	CS	CS
N40	Hyperplasia of prostate	CS	CS
R00.1	Bradycardia, unspecified	CS	CS
R01.1	Cardiac murmur, unspecified	CS	CS
R07	Pain in throat and chest	CS	CS
R07.0	Pain in throat	CS	CS
R13	Dysphagia	CS	CS
R16.0	Hepatomegaly, not elsewhere classified	CS	CS
R25.1	Tremor, unspecified	CS	CS
R30.0	Dysuria	CS	CS
R31	Unspecified haematuria	CS	CS
R51	Headache	CS	CS
R62.8	Other lack of expected normal physiological development	CS	CS
R63.0	Anorexia	CS	CS
S20.2	Contusion of thorax	CS	CS
S32.0	Fracture of lumbar vertebra	CS	CS
S62.6	Fracture of other finger	CS	CS
S82.8	Fractures of other parts of lower leg	CS	CS
S83.6	Sprain and strain of other and unspecified parts of knee	CS	CS
Z60.9	Problem related to social environment, unspecified	CS	CS
**Total**		198	100

Abbreviation: CS, Cell Suppression. Cells with fewer than 5 participants were suppressed to protect participant confidentiality.

© 2023 Hassan N et al. *JAMA Network Open*.
