# Supplementary material for: Sociodemographic Characteristics and Mental and Physical Health Diagnoses of Yazidi Refugees Who Survived the Daesh Genocide and Resettled in Canada
Source: JAMA Netw Open. 2023 Jul 12;6(7):e2323064. doi: 10.1001/jamanetworkopen.2023.23064 (PMC10339151; doi:10.1001/jamanetworkopen.2023.23064)
Supplement: Supplement 2. — Data Sharing Statement [file jamanetwopen-e2323064-s002.pdf]

Data Sharing Statement

Hassan. Sociodemographic Characteristics and Mental and Physical Health Diagnoses of Yazidi Refugees Who Survived the Daesh Genocide and Resettled in Canada. *JAMA Netw Open*. Published July 12, 2023. doi:10.1001/jamanetworkopen.2023.23064

Data
Data available: No

Additional Information
Explanation for why data not available: We are limited by our research agreement with the data custodian and ethics certificates by our institution which do not permit open data sharing. In these agreements, we are only permitted, upon request, to share a completely de-identified dataset with a journal to assist with verification of our analyses.
